# Predictive risk factors for pneumothorax after transbronchial biopsy using endobronchial ultrasonography with a guide sheath

**DOI:** 10.1186/s12890-021-01551-1

**Published:** 2021-05-29

**Authors:** Yusuke Gotoh, Teppei Yamaguchi, Hiroshi Yatsuya, Aki Ikeda, Takuya Okamura, Yosuke Sakakibara, Takuma Ina, Yuri Maeda, Mariko Hirochi, Hisashi Kako, Yasuhiro Goto, Sumito Isogai, Naoki Yamamoto, Masashi Kondo, Kazuyoshi Imaizumi

**Affiliations:** 1 Department of Respiratory Medicine, Fujita Health University School of Medicine, 1-98 Dengakugakubo, Kutsukake-cho, Toyoake, Aichi 470-1192 Japan; 2 Department of Thoracic Oncology, Aichi Cancer Center Hospital, 1-1 Kanokoden, Chikusa-ku, Nagoya, Japan; 3 Department of Public Health and Health Systems, Graduate School of Medicine, Nagoya University, 65 Tsurumai-cho, Nagoya, Japan; 4 Department of Ophthalmology, Kanazawa Medical University, Ishikawa, Japan

**Keywords:** Bronchoscopy, Complication, Fluoroscopy, Pneumothorax, Endobronchial ultrasonography, Transbronchial biopsy

## Abstract

**Background:**

Pneumothorax is one complication of transbronchial biopsy (TBB) using endobronchial ultrasonography with a guide sheath (EBUS-GS-TBB). We sought to clarify the risk factors for pneumothorax after EBUS-GS-TBB under fluoroscopic guidance.

**Methods:**

We retrospectively reviewed data from 916 patients who underwent EBUS-GS-TBB at Fujita Health University Hospital. We evaluated the following risk factors for pneumothorax after EBUS-GS-TBB: patient characteristics (sex, age, and pulmonary comorbidities); lesion data (location, size, existence of ground-glass opacities [GGOs], pleural involvement, computed tomography [CT] bronchus sign, visibility on fluoroscopy, and EBUS findings); final diagnosis; years of bronchoscopist experience; and guide sheath size. Univariate and multivariate logistic regression analyses were performed.

**Results:**

Among the 916 patients, 30 (3.28%) presented with pneumothorax. With a univariate analysis, factors that independently predisposed to pneumothorax included lesions containing GGOs, lesions in sagittal lung segments on fluoroscopy, lesions that were not visible on fluoroscopy, and infectious lesions. A univariate analysis also showed that lesions in the right upper lobe or left upper division, as well as malignant lesions, were less likely to lead to pneumothorax. Age, underlying pulmonary disease, CT bronchus sign, EBUS findings, bronchoscopist experience, and guide sheath size did not influence the incidence of pneumothorax. A multivariate analysis revealed that only lesions containing GGOs (odds ratio [OR] 6.47; 95% confidence interval [CI] 2.13–19.6, *P* = 0.001) and lesions in lung segments with a sagittal orientation on fluoroscopy (OR 2.47; 95% CI 1.09–5.58, *P* = 0.029) were significant risk factors for EBUS-GS-TBB-related pneumothorax.

**Conclusions:**

EBUS-GS-TBB of lesions containing GGOs or lesions located in sagittal lung segments on fluoroscopy correlate with a higher pneumothorax risk.

## Background

Since chest computed tomography (CT) screening for lung cancer has become a standard of care in routine clinical practice, there is an increasing need to diagnose small peripheral pulmonary lesions [1]. Transbronchial biopsy (TBB) using endobronchial ultrasound (EBUS) with a guide sheath (GS), referred to in this paper as EBUS‐GS-TBB, is a recently introduced advanced bronchoscopic technique that has facilitated a higher diagnostic yield for small peripheral lung lesions compared with conventional TBB [2, 3]. In general, TBB is a safe procedure for most patients. However, it sometimes leads to complications, including intrabronchial bleeding [4], infection [5], and pneumothorax [6], which can negatively affect the subsequent clinical course. Pneumothorax is one of the most common complications of TBB, with an incidence ranging from 1 to 4% [7]. Patients who develop pneumothorax may require chest tube drainage and prolonged hospitalization. In addition, for patients with respiratory insufficiency, pneumothorax may be serious or even fatal. Thus, preventing pneumothorax after TBB is vital. When using EBUS-GS-TBB, the precise location of the target lesion can be detected by ultrasound; however, this does not eliminate the possibility of pneumothorax [8]. Although previous studies have addressed the risk factors for pneumothorax after TBB with [9] or without [10] EBUS guidance, none have described the risk factors for pneumothorax after EBUS-GS-TBB under fluoroscopic guidance. The present study aimed to evaluate the incidence of, and risk factors for, pneumothorax after EBUS-GS-TBB under fluoroscopic guidance.

## Methods

### Patients and data acquisition

This study was a single-center retrospective review. The medical records of patients who underwent EBUS-GS-TBB for peripheral solitary lung lesions between August 2013 and March 2017 at Fujita Health University Hospital were examined. Peripheral pulmonary lesions were defined as lesions surrounded by lung parenchyma without direct visualization by bronchoscopy. Patients who underwent biopsy for ≥ 2 separate lesions were excluded. We investigated the incidence of pneumothorax after EBUS-GS-TBB, sequential therapy for pneumothorax, and resulting outcomes. We also reviewed the following data of all patients: age; sex; smoking status; lung complications (emphysema, fibrosis, postoperative changes, and other lung diseases); steroid use; thin-section CT images of target lesions (size, presence of ground-glass opacities [GGOs], location, CT bronchus sign [3], presence of pleural involvement); EBUS findings (within, adjacent to, or outside) [2]; bronchoscopic diagnosis; and guide sheath size. This study was approved by the institutional review board of Fujita Health University (HM-19-017), and the study was performed in accordance with the principles of the Declaration of Helsinki.

### Bronchoscopic procedure

All patients provided written informed consent before undergoing bronchoscopy. All patients underwent thin-section CT (0.5-mm slice thickness) within 1 month prior to the procedure. Each bronchoscopist evaluated the CT images and identified the bronchus and target lesion [3]. The responsible bronchus and the characteristics of target lesions were routinely confirmed by discussion between more than two bronchoscopists. A virtual navigation system was used in eight cases, all of which had a history of lobectomy (post-surgery). Pharyngeal local anesthesia with 2% lidocaine was administered prior to bronchoscopy. All patients received moderate sedation with midazolam at an individualized dose [11], and an additional intramuscular injection of 30 mg of pethidine hydrochloride was administered to patients aged < 65 years.

In total, 25 bronchoscopists performed EBUS-GS-TBB in the study period. The number of years of clinical experience ranged from 2 to 30 years (median, 5 years). We routinely perform bronchoscopy with the involvement of at least three bronchoscopists (one operator, one assistant, and one who monitors the patient’s general condition). Five-hundred twenty-seven procedures (57.7%) were performed by bronchoscopists with fewer than five years of experience in bronchoscopy.

EBUS-GS was performed using an endoscopic ultrasound system (EU-M30S, Olympus Co. Ltd., Tokyo, Japan) with a 20-MHz radial-type ultrasound probe (UM-S20-20R, Olympus Co. Ltd.) according to the standard Kurimoto method [2]. We used two types of guide sheath kit (a large kit [K-203, 2.55-mm outer diameter guide sheath equipped with 1.9-mm outer diameter biopsy forceps] or a small kit [K-201, 1.95-mm outer diameter guide sheath equipped with 1.5-mm outer diameter biopsy forceps]) (Olympus Co. Ltd.) in combination with a UM-S20-20R probe. Flexible fiberoptic bronchoscopes used in this study were BF-260, BF-P260F, BF-P290, BF-1T260, and BF-1TQ290 (Olympus Co. Ltd.). We routinely obtained 12 biopsy samples with a small guide sheath kit (K201) and 6–9 samples with a large guide sheath kit (K203). TBB was routinely assisted by X-ray fluoroscopy (Canon Medical Systems, Tokyo, Japan). Blood pressure, heart rate, and oxygen saturation were continuously measured during bronchoscopy.

### Target lesion data analysis

All target lesion imaging data were collected from CT images with a 0.5-mm slice thickness generated using a non-enhanced multidetector CT system (Aquilion One Vision Edition; Toshiba Medical Systems, Tokyo, Japan) within 1 month before bronchoscopy. We collected data on the size, location, characteristics (GGOs vs. solid), CT bronchus sign (A–C, with A being the responsible bronchus that clearly reached the inside of the target lesion and C being no detectable responsible bronchus. If neither type A nor type C was concluded, the CT bronchus sign was categorized as type B) [3], existence of pleural involvement (contact or indentation), and GGO-dominant nodules (consolidation-to-tumor ratio [CTR] of < 0.5) designated as ground-glass nodules [GGNs]) [12]. In this study, we defined lung segments with a sagittal orientation on fluoroscopy (right: S2a, S3b, S5ab, S6a, S6c, S10a, S10c; left: S1 + 2b, S3b, S5ab, S6a, S6c, S10a, S10c) as “lung segments in a sagittal direction.” We focused on these segments because we could not easily recognize the pleural edge in the sagittal direction under fluoroscopic guidance. Thus, the positional relationship between the biopsy forceps and the pleura was difficult to recognize.

### Diagnosis and management of pneumothorax

All patients underwent chest X-ray immediately following bronchoscopy and on the day after bronchoscopy. When pneumothorax was diagnosed, attending clinicians selected tube drainage or conservative management (observation) according to the extent of lung collapse and each patient’s symptoms. If the apex of a collapsed lung was above the clavicle on chest X-ray (posteroanterior [PA] upright position) and the patient did not exhibit significant dyspnea, clinical observation was often selected; however, in some cases, chest drainage and prolonged hospitalization were used. Oxygen supplementation was administered based on the attending clinician’s judgment.

### Statistical analysis

To determine the risk factors for pneumothorax after EBUS-GS-TBB, we investigated the clinical factors related to pneumothorax after bronchoscopy, including age (> 75 years), sex, pulmonary comorbidities (severe emphysema and pulmonary fibrosis), lesion location, fluoroscopic view (not visible), lesion size (long diameter of < 20 mm), GGNs (CTR of > 0.5), CT bronchus sign (CT bronchus sign B or C), pleural contact/indentation, EBUS findings (other than “within”), bronchoscopists’ clinical experience, guide sheath size, and final diagnosis (malignancy and infectious disease). Associations between the variables of interest and the incidence of pneumothorax after EBUS-GS-TBB were first examined by univariate logistic regression and presented as odds ratios (ORs) and 95% confidence intervals (CIs). Next, multivariate logistic regression for pneumothorax was performed using all variables that were used for the univariate analysis. A backward variable selection procedure was employed to obtain the final model. We presented mutually adjusted ORs and 95% CIs for pneumothorax after EBUS-GS-TBB for the final model. We also conducted stratified analyses according to lesion location; that is, lung segments in the sagittal direction, which are difficult to recognize under fluoroscopic guidance, or other subsegments. All statistical analyses were performed using JMP version 15.2 statistical software (SAS Institute Inc., Tokyo, Japan).

## Results

### Patient and target lesion characteristics

During the study period, 921 patients underwent EBUS-GS-TBB. Five patients were excluded from the study because they underwent biopsy of ≥ 2 lesions in different lung lobes. Thus, 916 patients were included in the study. Thirty patients (3.28%) developed pneumothorax after EBUS-GS-TBB, which was resolved in 22 patients by observation. Eight patients required chest drainage, and four patients developed respiratory failure (SpO_2_ < 90%) when pneumothorax occurred. All patients recovered without any surgical intervention or pleurodesis. None of the patients experienced pneumothorax recurrence. Patients’ characteristics are shown in Table 1. The median age of patients was 72 years (range, 23–91 years). A total of 236 patients (25.7%) had confluent or advanced destructive emphysema [13], and 129 patients (14.1%) had pulmonary fibrosis. In 759 cases (82.9%), we used a small guide sheath kit (K201).

**Table 1:** Characteristics of patients

Characteristics	Total	No pneumothorax	Pneumothorax
	n = 916	n = 886	n = 30
Age			
Median (IQR)	72 (65–77)	71 (65–77)	71 (60–74)
≥ 75 years	325 (35.2%)	313 (35.3%)	11 (36.7%)
Sex			
Male	609 (66.5%)	590 (66.6%)	19 (63.3%)
Female	307 (33.5%)	296 (33.4%)	11 (36.7%)
Smoking status			
Current	230 (25.1%)	222 (25.2%)	8 (26.7%)
Ex	628 (68.6%)	608 (68.5%)	13 (43.3%)
Never	58 (6.3%)	56 (6.3%)	9 (30.0%)
Pulmonary co-morbidities			
Emphysema*	236 (25.8%)	227 (25.6%)	10 (33.3%)
Fibrosis	129 (14.1%)	122 (13.9%)	7 (23.3%)
Post-surgery	24 (2.6%)	24 (2.7%)	0 (0%)
Other lung diseases**	88 (9.6%)	83 (9.4%)	5 (16.7%)
Steroid use	35 (3.8%)	35 (3.9%)	0 (0%)
Guide sheath used			
Large (outer diameter 2.55 mm)	157 (17.1%)	155 (17.4%)	3 (10.0%)
Small (outer diameter 1.95 mm)	759 (82.9%)	731 (82.5%)	27 (90.0%)

^*^Confluent emphysema and advanced destructive emphysema [13]

^**^Other lung diseases, including bronchial asthma, sequelae of tuberculosis, bronchiectasis, sarcoidosis, pulmonary non-*Mycobacterium tuberculosis* infection, and pneumoconiosis

Table 2 shows data on target lesions. The median lesion size was 23.13 mm (range, 5.5–112.8 mm), and 52 lesions (5.6%) were GGNs [12]. A total of 234 lesions (23.4%) were present in sagittal lung subsegments. Lesions located in these segments were difficult to recognize under fluoroscopic guidance. The CT bronchus signs of 518 lesions (56.5%) were positive, which indicated that the responsible bronchus clearly reached the inside of the target lesion [3]. EBUS findings of target lesions showed “within” in 558 cases (60.9%). Final target lesion diagnoses were as follows: lung cancer or other malignancy (651 cases [71.1%]), infectious disease (56 cases [6.1%]), other inflammatory disease (121 cases [13.2%]), and other benign diseases (88 cases [9.6%]). The total diagnostic yield of EBUS-GS-TBB in this study was 86.6%.

**Table 2:** Data on target lesions

	Total	No pneumothorax	Pneumothorax
	n = 916	n = 886	n = 30
Lesion size			
Median (IQR) mm	23.08 (17.1–32.8)	23.13 (17.1–32.8)	20.38 (17.4–33.2)
< 20 mm (long diameter)	338 (36.9%)	325 (36.7%)	13 (43.3%)
Findings of thin-section CT			
GGN*	53 (5.8%)	47 (5.3%)	6 (20.0%)
Solid	863 (94.2%)	839 (94.5%)	24 (80.0%)
Location			
Right upper lobe or left upper segments	448 (48.9%)	440 (49.7%)	8 (26.7%)
Middle lobe or lingula	127 (13.9%)	119 (13.4%)	8 (26.7%)
Lower lobe	341 (37.2%)	327 (36.9%)	14 (46.7%)
Segments located in the sagittal direction**	214 (23.4%)	201 (22.7%)	13 (43.3%)
CT bronchus sign^***^			
A	518 (56.7%)	503 (56.7%)	15 (50.0%)
B	336 (36.7%)	323 (36.5%)	13 (43.3%)
C	62 (6.8%)	60 (6.8%)	2 (6.7%)
Pleural involvement			
Contact or indentation	344 (37.5%)	333 (37.6%)	11 (36.7%)
No contact	572 (62.5%)	553 (62.4%)	19 (63.3%)
Fluoroscopic view			
Visible	643 (70.2%)	627 (70.8%)	16 (53.3%)
Not visible	273 (29.8%)	259 (29.2%)	14 (46.7%)
EBUS findings			
Within	558 (60.9%)	543 (61.2%)	15 (53.3%)
Adjacent to	236 (25.8%)	227 (25.6%)	9 (30.0%)
Blizzard sign and mixed-blizzard sign	97 (10.5%)	92 (10.3%)	5 (16.7%)
Outside	25 (2.7%)	24 (2.7%)	1 (3.3%)
Bronchoscopic diagnosis			
Malignancy	647 (70.6%)	633 (71.4%)	14 (46.7%)
Infection	55 (6.0%)	50 (5.6%)	5 (16.7%)
Organizing pneumonia	45 (4.9%)	45 (5.1%)	0 (0.0%)
Other inflammatory lesions	61 (6.7%)	60 (6.7%)	1 (3.3%)
Undiagnosed	108 (11.8%)	98 (12.1%)	10 (33.3%)
Final diagnosis			
Lung cancer or other malignancy	651 (71.1%)	634 (71.6%)	17 (56.7%)
Infectious disease	56 (6.1%)	51 (5.7%)	5 (16.7%)
Inflammatory disease	121 (13.2%)	116 (13.1%)	5 (16.7%)
Other benign disease	88 (9.6%)	85 (9.6%)	3 (10.0%)

*Abbreviations*: CT, computed tomography; EBUS, endobronchial ultrasonography

^*^GGN, ground-glass-opacity-predominant nodule (consolidation-to-tumor ratio of < 0.5)

^**^ Lung segments that present with a sagittal orientation on fluoroscopy (right: S2a, S3b, S5ab, S6a, S6c, S10a, S10c; left: S1 + 2b, S3b, S5ab, S6a, S6c, S10a, S10c)

^***^ CT bronchus sign: type A (the responsible bronchus clearly reached the inside of the target lesion); type C (no bronchus could be detected in relation to the lesion); or type B (neither type A nor type C) [3]

### Predictive factors for pneumothorax

The univariate logistic regression analysis revealed that lesions located in lung segments in a sagittal direction, lesions with GGN features, lesions that were not visible on fluoroscopy, and infectious lesions were prone to EBUS-GS-TBB-related pneumothorax. The univariate analysis also indicated that EBUS-GS-TBB for lesions located in the right upper lobe or left upper division, as well as malignant lesions, were less likely to lead to pneumothorax (Table 3). The number of years of clinical experience of each bronchoscopist did not influence the incidence of pneumothorax. The multivariate analysis revealed that GGN features (OR 6.47, 95% CI 2.13–19.6, *P* = 0.001) and lesions located in sagittal lung segments (OR 2.47, 95% CI 1.09–5.58, *P* = 0.029) were significantly associated with EBUS-GS-TBB-related pneumothorax (Table 3).

**Table 3:** Univariate and multivariate odds ratios (ORs) and 95% confidence intervals (95% CIs) of potential risk factors for EBUS-GS-TBB-related pneumothorax

	Univariate analysis			Multivariate analysis
	OR (95% CI)	P value	OR (95% CI)	P value
Age (≥ 75 years)	1.07 (0.50–2.27)	0.848		
Sex (male)	0.87 (0.41–1.84)	0.698		
Pulmonary complication				
Emphysema (severe emphysema*)	1.46 (0.67–3.17)	0.395	2.13 (0.47–2.12)	0.074
Pulmonary fibrosis	1.91 (0.80–4.54)	0.175		
Lesion location				
Upper lobe (Rt) or upper segment (Lt)	0.37 (0.16–0.84)	0.015	0.38 (0.14–1.01)	0.053
Lingula or middle lobe	2.39 (1.04–5.49)	0.052	1.46 (0.45–2.99)	0.358
Lower lobe	1.49 (0.72–3.10)	0.337		
Segments located in the sagittal direction**	2.61 (1.24–5.46)	0.014	2.47 (1.09–5.58)	0.029
Lesion appearance				
Long diameter (< 20 mm)	1.32 (0.63–2.75)	0.449		
GGN*** (vs. solid lesion)	4.46 (1.74–11.44)	0.006	6.47 (2.13–19.6)	0.001
CT bronchus sign B or C ^ref XX^	1.31 (0.63–2.72)	0.461		
Pleural contact or indentation	0.96 (0.45–2.04)	0.961		
EBUS finding other than “within”	1.39 (0.67–2.87)	0.450		
EBUS finding “blizzard”/ “mixed-blizzard”	1.96 (0.73–5.26)	0.195		
Not visible on fluoroscopy	2.11 (1.01–4.40)	0.043	1.46 (0.65–3.28)	0.358
Final diagnosis of target lesions				
Malignancy	0.46 (0.22–0.96)	0.042	0.64 (0.66–3.67)	0.312
Infectious disease	3.34 (1.23–9.11)	0.029	3.28 (0.99–10.87)	0.051
Years of bronchoscopist experience				
Fewer than 5 years	1.27 (0.60–2.73)	0.510		
Guide sheath				
Large (diameter 2.6 mm)	1.35 (0.45–3.31)	0.562		

^*^Confluent or advanced destructive emphysema [14]

^**^Right: S2a, S3b, S5a, S5b, S6a, S6c, S10a, S10c; left: S1 + 2b, S3b, S5a, S6a, S6c, S10a, S10c

^***^GGN: ground-glass-opacity-predominant nodule (consolidation-to-tumor ratio of < 0.5)

*Abbreviations*: CT, computed tomography; EBUS, endobronchial ultrasonography

Fluoroscopic recognition of target lesions depends on the imaging performance of the fluoroscopic system. For example, fluoroscopy with a C-arm or cone-beam CT could offer more recognizable images, even at lung segments in the sagittal direction. Thus, we further investigated whether the risk factors for EBUS-GS-TBB-related pneumothorax changed when lesion location was excluded from the analysis. A multivariate analysis of patients whose lesions were located in sagittal lung subsegments (n = 214) and patients whose lesions were present in other subsegments (n = 702) revealed that only GGNs were significant risk factors for EBUS-GS-TBB-related pneumothorax in both groups (OR 6.43, 95% CI 1.89–21.89, *P* = 0.003 and OR 5.02, 95% CI 1.42–17.76, *P* = 0.002, respectively).

## Discussion

The present study demonstrates that the risk factors for EBUS-GS-TBB-related pneumothorax are GGOs in target lesions and lesions located at lung subsegments in a sagittal direction.

GGO-dominant lesions were difficult to detect using fluoroscopy [14, 15]. EBUS recognition for GGO-dominant lesions is also difficult because ultrasound images of such lesions are sometimes vague or unrecognizable [12]. In fact, recent reports have evaluated the diagnostic yield of EBUS-GS-TBB for the diagnosis of GGNs, which ranged from 57 to 66% [12, 16, 17]. This is lower than the diagnostic yield of EBUS-GS-TBB for lesions that present as solid nodules or with consolidation [12]. Izumo et al. reported the “blizzard sign or mixed-blizzard sign” as the specific EBUS finding for GGN lesions [14]. However, as these EBUS findings are subtle, it is difficult to confirm the precise location of GGNs using either EBUS or fluoroscopy. We speculate that difficulty in both fluoroscopic and EBUS recognition correlates with a significant increase in the occurrence of pneumothorax in GGN lesions.

Pneumothorax during TBB is caused by injury of the visceral pleura by biopsy forceps or bronchial brushes [18]. The tips of biopsy forceps or guide sheaths are difficult to recognize at subsegments in the sagittal direction when using fluoroscopy (anterior–posterior view). With conventional TBB without an EBUS guide, fluoroscopic guidance is essential to perform TBB of peripheral lung nodules. Although the EBUS system can directly detect peripheral lung lesions, it is impossible to perform biopsy with real-time ultrasound guidance in EBUS-GS-TBB. Thus, fluoroscopy is essential to confirm the positional relationship of guide sheaths or biopsy forceps with the pleura. These data indicate that fluoroscopy during TBB is important, even with the EBUS guide, not only for a higher diagnostic yield, but also for procedural safety. More importantly, because most patients who undergo bronchoscopy are mildly sedated, it is difficult for the operator to recognize pleural pain, which may also be related to pneumothorax onset [19, 20]. Thus, careful fluoroscopic observation during TBB is important, even with EBUS guidance.

Our study also showed that EBUS-GS-TBB for lesions in the upper lobe or upper division of the left upper lobe is unlikely to be accompanied by pneumothorax. With real-time fluoroscopic guidance, movement of lesions by breathing was less pronounced in the upper lung field compared with the lower lung field. Thus, lesions in the upper lobe and upper division may be easier to recognize during bronchoscopy under fluoroscopic guidance, which is beneficial for avoiding iatrogenic pneumothorax.

Huang et al. reported predictive risk factors for pneumothorax after EBUS-guided TBB [9]. They included pulmonary emphysema around target lesions and EBUS findings “adjacent to the lesion,” both of which differ compared with our results. We speculate that the reason for these discrepancies is that Huang et al. did not use a guide sheath system in their study. The guide sheath system used in the present study is widely used because repeated biopsies from the planned point can be performed through the sheath placed on or near the target lesion. Use of a guide sheath and careful observation of EBUS findings could prevent unnecessary injury of emphysematous lung tissue. Another study showed that TBB from the upper lobes is significantly associated with post-TBB pneumothorax, which is different from our result [10]. However, this study included patients who underwent TBLB for diffuse lung lesions. Moreover, fluoroscopic guidance was not used in this study, and the incidence of post-TBB pneumothorax was higher compared with our study (6% vs. 3.28%, respectively).

A recent study showed that even with newly developed robotic bronchoscopy, 3.7% of patients are complicated with pneumothorax [21]. This report and our study suggest that it is difficult to completely prevent pneumothorax, even when TBB is performed using an advanced tool or technique. Thus, rapid and accurate diagnosis, as well as adequate and prompt therapy to treat pneumothorax, must be performed after TBB. Even with EBUS-GS support, TBB should be performed with caution in patients with identified risk factors. Some new modalities, such as cone-beam CT, may be beneficial for EBUS-GS-TBB of lesions at lung segments in the sagittal direction.

One limitation of our study is that it is a single-center study with a retrospective design. Bronchoscopy procedures, including biopsy, sedation, and fluoroscopy, vary between institutes. Thus, multi-institutional prospective studies are warranted in the future. Nevertheless, we believe our study reveals solid risk factors for pneumothorax after EBUS-GS-TBB, since these risk factors were determined from a large number of patients.

## Conclusions

Our study shows that with EBUS-GS-TBB, we should be aware of the risk of pneumothorax when lesions are located in sagittal lung segments on fluoroscopy, because the positional relationship between biopsy forceps and the pleura is difficult to recognize, even under EBUS guidance. The risk of pneumothorax should also be considered when lesions present as GGOs, which are difficult to identify, even in combination with fluoroscopy and EBUS.

## Data Availability

The datasets generated and analyzed during the current study are available from the corresponding author on reasonable request.

## Abbreviations

CI: Confidence interval
CT: Computed tomography
CTR: Consolidation-to-tumor ratio
EBUS-GS: Endobronchial ultrasonography with a guide sheath
GGN: Ground-glass nodule
IQR: Interquartile range
OR: Odds ratio
PA: Posteroanterior
TBB: Transbronchial biopsy
